# Normobaric hypoxic conditioning in men with metabolic syndrome

**DOI:** 10.14814/phy2.13949

**Published:** 2018-12-18

**Authors:** Lars Klug, Anja Mähler, Natalia Rakova, Knut Mai, Jeanette Schulz‐Menger, Gabriele Rahn, Andreas Busjahn, Jens Jordan, Michael Boschmann, Friedrich C. Luft

**Affiliations:** ^1^ Experimental & Clinical Research Center (ECRC) a joint collaboration between Max‐Delbrück Center for Molecular Medicine and Charité Universitätsmedizin Berlin Germany; ^2^ Department of Endocrinology & Metabolism Charite Universitätsmedizin Berlin Berlin Germany; ^3^ Clinical Research Unit Berlin Institute of Health (BIH) Berlin Germany; ^4^ DZHK (German Centre for Cardiovascular Research), partner site Berlin Berlin Germany; ^5^ Department of Cardiology and Nephrology HELIOS Klinikum Berlin Buch Berlin Germany; ^6^ Present address: Institut für Luft‐ und Raumfahrtmedizin Deutsches Zentrum für Luft‐ und Raumfahrt (DLR) Köln Germany

**Keywords:** Exercise training, hypertension, metabolic syndrome, normobaric hypoxia

## Abstract

The evidence that physical exercise lowers metabolic and cardiovascular risk is undisputed. Normobaric hypoxia training has been introduced to facilitate the effects of exercise. We tested the hypothesis that hypoxia training augments exercise‐related effects. We randomized 23 men with metabolic‐syndrome to single‐blinded exercise at normoxia (FiO_2_ 21%) or hypoxia (FiO_2_ 15%). Six weeks endurance training on a treadmill, 3 days per week, over 60 min at 60% *V*O_2_max was required. The study included the following: (1) metabolic phenotyping by indirect calorimetry and adipose and muscle tissue microdialysis to gain insight into effects on resting, postprandial, and exercise metabolism, (2) cardiac imaging, and (3) biopsies. Primary endpoint was the change in cardiorespiratory fitness; secondary endpoints were as follows: changes in body weight, waist circumference, blood pressure, cardiac dimensions, and adipose and muscle tissue metabolism and gene expression. Our subjects reduced waist circumference and improved several cardiovascular risk markers including blood pressure. However, these effects were similar in both training groups. Cardiac dimensions were not influenced. We focused on glucose metabolism. After an oral glucose load, adipose tissue metabolism was significantly shifted to a more lipolytic state under hypoxia, whereas muscle metabolism was similar under both conditions. Postprandial energy expenditure was significantly increased under hypoxia, whereas activity energy expenditure was improved under normoxia. Gene expression was not consistently influenced by FiO_2_. Adipose tissue triglyceride lipase, leptin, and hypoxia‐inducible factor‐alpha expression were increased by normoxia but not hypoxia.

## Introduction

In a recent *clarion call*, the Global Burden of Metabolic Risk Factors for Chronic Diseases Collaboration emphasized that the cardiometabolic disease and risk factor epidemic at the beginning of the 21st century are high blood pressure and an increasing effect of obesity and the metabolic syndrome leading to type‐2 diabetes (Global Burden of Metabolic Risk Factors for Chronic Diseases C, 2014). The group pointed out that lowering cardiometabolic risks through dietary, behavioral, and pharmacological interventions should be a part of the global response to non‐communicable diseases. The diagnosis of the metabolic syndrome requires abnormalities in three or more of the Adult Treatment Panel III of the National Cholesterol Education Program (NCEP ATP III) criteria, which include the following: a waist circumference of more than 102 cm in men and more than 88 cm in women, a triglyceride level of ≥150 mg/dL, a level of high‐density lipoprotein (HDL) cholesterol <40 mg/dL in men and <50 mg/dL in women, a blood pressure >130/85 mm Hg or taking antihypertensive drugs, and a fasting glucose level ≥110 mg/dL, albeit waist circumference is the most facile screening tool (Alberti et al. 2005). The pathogenesis of the metabolic syndrome is thought to involve a complex interaction of multiple factors, which include obesity, abnormal fat distribution, insulin resistance, hepatic, vascular, and immunologic factors, as well as lifestyle and genetic contributions (Grundy et al. 2004). Physical exercise has great appeal as a treatment (Lear et al. 2017). Exercise is arduous and shortcuts to success would be welcome. Normobaric hypoxic conditioning is defined as exposure to systemic and/or local hypoxia at rest (passive) or combined with exercise training (active) (Workman and Basset 2012). Hypoxic conditioning has been advocated as an aid to healthy and athletic populations to improve their performance. Our group has conducted two controlled studies of hypoxic training in subjects without metabolic abnormalities that gave encouraging results (Haufe et al. 2008; Wiesner et al. 2010). We recognize the importance of translating our notions to a human disease. We, therefore, now tested the hypothesis that a similar study in male subjects with the metabolic syndrome would show benefit of hypoxia training.

## Methods

The study was registered with ClinicalTrials.gov, number NCT 01468220. The Charité Ethical Committee on human subjects approved the study and written informed consent was obtained from all participants. We hypothesized that normobaric hypoxia training would augment fat loss in response to exercise, not only by affecting exercise energy expenditure, but also by influencing exercise training‐induced changes in respiratory exchange ratio (RER) at rest.

### Patients

**Figure 1:**
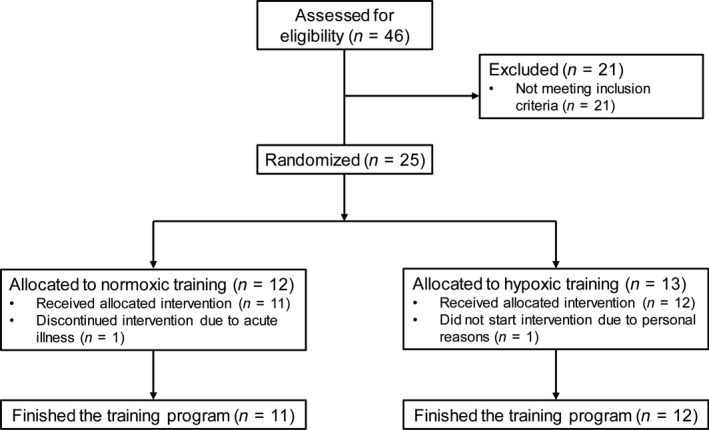
Subjects and course of the study.

Our rationale was based on the idea that if the hypoxia‐training idea is of value, it should show benefit in a human disease. We included 23 men with metabolic syndrome according to the NCEP ATP III definition (last follow‐up April 2016) in our study (Fig. 1). These subjects were invariably non‐exercising adults who did not engage in planned, organized physical activity. All subjects completed a comprehensive medical evaluation. Key inclusion criteria were: age between 18 and 70 years and the presents of the metabolic syndrome defined in accordance to the NCEP ATP III criteria (see above). Key exclusion criteria were as follows: walking disability, clinical relevant acute or chronical inflammatory diseases and alcohol or drug abuses. Subjects were advised to continue their current physical activity level throughout the study. Other than generalized health‐care advice, we did not counsel the subjects further.

### Study design

We designed a prospective, randomized, controlled study conducted in an academic clinical research center, which compared the effects of a moderate 12‐week endurance exercise training on a treadmill under either normoxia or hypoxia (both normobaric) on cardiometabolic risk factors. Subjects underwent thorough anthropometric, metabolic, and exercise testing before and after the respective training program. For allocation of the subjects, a computer‐generated list of random numbers was used. The randomization sequence was created using SPSS 18 (SPSS, Inc., Chicago, IL, USA) statistical software and subjects were assigned to an exercise training program under either normoxic (FiO_2_ 21%) or hypoxic (FiO_2_ 15%) conditions (both normobaric) with a 1:1 allocation using random block sizes of 2, 4, and 6. Patients were blinded to the training conditions while the person who supervised the training session was not. We are aware of the fact that simulated altitude and terrestrial altitude are not necessarily the same. The study protocol, detailed training intervention, the hypoxia chamber, indirect calorimetry methodology, microdialysis, biopsy technique is described in detail below.

### Study protocol

After entry into the study, patients underwent a detailed cardiorespiratory and metabolic evaluation on three consecutive days before and after the six‐weeks training program. On day 1, patients arrived in our study center at 8:00 AM after a 12‐h overnight fast. At the day before, patients were asked to abstain from (1) any kind of physical activity, (2) caffeine and alcohol containing beverages, and (3) from smoking.

Body composition was assessed by bioelectrical impedance analysis (BIACorpus RX Spectral, MEDICAL Healthcare GmbH, Karlsruhe, Germany). Then resting and postprandial energy expenditure were measured before and after an oral glucose load, respectively (300 mL solution, 75 g glucose load, ACCUCHEK ^®^ Dextro^®^, O.G.T., Hoffmann‐La Roche AG, Grenzach‐Wyhlen Germany). Systemic energy metabolism was studied by indirect calorimetry (canopy system) and by collecting venous blood samples from a large antecubital vein. Local tissue metabolism was studied by microdialysis of the abdominal subcutaneous adipose tissue (aSAT) and skeletal muscle (*Vastus lateralis*) before and after the glucose load.

On day 2, patients arrived again at 8:00 AM after a 12‐h overnight fast in our study center. Body composition was assessed by air displacement plethysmography (BodPod, COSMED Deutschland GmbH, Fridolfing, Germany). Then, patients were placed in our respiratory chamber in order to monitor $\dot{V}$O_2_ consumption and $\dot{V}$CO_2_ production at rest (30 min) and during moderate bicycle exercise (60 min) on a bicycle ergometer (VIASprint 150 P, Ergoline, Bitz, Germany). Exercise was performed at a pre‐defined workload determined by a maximum work‐load test of 50%*V*O_2_max, followed by a 40‐min recovery period. $\dot{V}$O_2_ consumption and $\dot{V}$CO_2_ production were used to calculate resting and exercise activity energy expenditure (EE) and respiratory exchange ratio (RER, *V*CO_2_/*V*O_2_). During exercise, heart rate and O_2_‐saturation were continuously monitored and rates of perceived exertion on a 10‐point scale were recorded every 10 min. After the exercise program, patients had a 2 h break for a standardized lunch. Then, cardiorespiratory fitness was tested by an incremental cycle ergometer test (Sana cardio 250 SE, Ergosana, Bitz, Germany). Endpoint was patient‐determined failure to continue because of exhaustion. The test started with a 1‐min rest to determine baseline parameters followed by a 2‐min warm‐up at 20 W. Initial exercise work‐load was set to 25 W followed by a stepwise increase in 25 W every 3 min until patient's exhaustion or meeting discontinuation criteria. After the test, there was a 5‐min recovery period without workload. During the test, breath‐by‐breath gas exchange and an electrocardiogram were recorded (Quark RMR, COSMED, Italy). At the end of every incremental step, blood samples were taken from an earlobe in order to analyze lactate. On day 3, abdominal subcutaneous tissue and skeletal muscle (*Vastus lateralis*) biopsies were obtained.

### Training intervention

Patients completed a moderate, aerobic exercise program over 6‐weeks/three times a week on a motorized treadmill (mercury 4.0, h/p/cosmos sports & medical GmbH, Germany) at 50–60% of their individual maximal heart rate, which was determined by the incremental cycle ergometer test. One training session lasted 60 min with three 15 min intervals of walking on the treadmill and a 5 min break in between for recovery. The intervention group trained under normobaric hypoxia (15% FiO_2_) simulating an altitude of 2500 m, while the control group trained under normobaric normoxia (21% FiO_2_). Individual training heart rate was adjusted by changing treadmill slope and/or pace by the supervisor and was – similarly to the O_2_ saturation – monitored continuously throughout the entire session.

### Hypoxia chamber

Trainings sessions took place in a normobaric hypoxia chamber (11 m², 38 m³, Linde AG, Berlin, Germany). The oxygen content within the chamber was reduced by mixing the incoming air with nitrogen. Throughout the trainings sessions, chamber O_2_ and CO_2_ concentrations were controlled by two independent sensors each (HTK, Hamburg, Germany). Depending on the measured gas concentrations, the inflow of fresh air and nitrogen was automatically adjusted by the system.

### Indirect calorimetry

We assessed changes in VO_2_ consumption and VCO_2_ production by indirect calorimetry in order to calculate changes in EE, RER, and carbohydrate and fat oxidation rates. For measuring these parameters at rest and after a glucose load, we used a canopy system (Quark RMR, COSMED Deutschland GmbH, Fridolfing, Germany). For measuring these parameters before, during and after bicycle exercise, we used a metabolic chamber. This chamber is a comfortable, airtight room (width: 2.5 m, depth: 2.0 m, height: 2.2 m, 11 m³) that is constantly supplied with fresh air like an open‐circuit indirect calorimeter. Changes in $\dot{V}$O_2_ consumption and $\dot{V}$CO_2_ production were measured, while patients were seated in a comfortable chair at rest or exercising on a bicycle and listening music or watching TV.

### Microdialysis

Microdialysis is a minimal‐invasive technique for monitoring the dynamics in tissue perfusion and metabolism. We inserted one microdialysis probe (M71, molecular cut‐off 100 kDa, *μ*Dialysis, Stockholm, Sweden) into abdominal subcutaneous adipose tissue and one into skeletal (*Vastus lateralis*) muscle. Before probe insertion, tissues were anaesthetized by subcutaneous 2% lidocaine injections. After probe insertion, probe perfusion was started with lactate‐free Ringer's solution (+ 50 mmol/L ethanol) at a flow rate of 2 *μ*L/min using microdialysis pumps (M102, *μ*Dialysis, Stockholm, Sweden). Ethanol was added to the perfusate to assess changes in tissue perfusion using the ethanol dilution technique (Boschmann et al. 2010). Dialysates were sampled in 15‐min intervals and analyzed for (1) ethanol by spectrophotometry (Boschmann et al. 2010) and (2) marker metabolites such as glucose, lactate and pyruvate (glucose supply and metabolism), and glycerol (lipid mobilization) by colorimetric assays (ISCUS^*flex*^, *μ*Dialysis, Stockholm, Sweden).

### Biopsies

Biopsies from abdominal subcutaneous adipose tissue and skeletal muscle (*Vastus lateralis*) were taken at baseline and after 6 weeks of the training intervention. The procedures were done immediately after the last exercise session. Before the biopsies, the respective skin areas were anesthetized with 2% lidocaine without epinephrine. Subsequently, a skin incision (3–4 mm) was made. Muscle biopsies were taken by repeated needle biopsies (Bergström needle). Adipose tissue samples (app. 2.0 g) were obtained by needle biopsies from the periumbilical region using an aspiration through a conventional 12 G needle. Both fat and muscle samples were snap‐frozen in liquid nitrogen and stored at −80°C until further analysis.

### Tissue preparation and real‐time quantitative polymerase chain reaction

Frozen tissue was homogenized and total RNA from human muscle tissue was isolated according to the manufacturer's instructions of SV Total RNA Isolation (Promega, Mannheim, Germany). RNA samples were stored at −80°C until assayed.

Complementary DNA (cDNA) synthesis was done according to manufacture manual (High Capacity RNA‐tocDNA Kit; Applied Biosystems, Foster City, CA). Samples were analyzed in triplicate with Power SYBR Green PCR Master Mix (Applied Biosystems). Real‐time quantitative polymerase chain reaction was performed using an ABI PRISM 7300 System (using SDS 1.4 system software, Applied Biosystems). The expression level of cyclophyllin A was used as an internal control. Primer sequences of analyzed genes are available on request. Cycle threshold values were used to calculate the amount of amplified polymerase chain reaction product in comparison to the housekeeping gene cyclophyllin A. The relative amounts of each transcript were analyzed using the 2^−ΔC(t)^ method.

### Cardiac magnetic resonance imaging

All CMR scans were performed on a clinical 1.5 Tesla MR scanner (Avanto, Siemens Medical Solutions AG, Erlangen, Germany) using a 12‐channel cardiac array coil. We performed cine imaging with a standard steady‐state free precession sequence to assess cardiac structure and function. We acquired three long axes and a complete short‐axis package covering the left ventricle from base to apex during repetitive breath holds in end‐expiration (Toka et al. 2015).

### Statistical analysis

The endpoints were compared according to distribution characteristics by either t‐test or Wilcoxon‐test for independent or repeated measures as appropriate. We intended to include 16 patients (eight males, eight females) per training group (normoxic or hypoxic). However, we had substantial difficulty convincing women to participate. Thus, we recruited 23 men with metabolic syndrome who were randomized to treatments. The endpoints were compared with analysis of variance, repeated measures as indicated and adjusted *T* tests. Fiducial limits are mean ± standard error. A value of *P* < 0.05 was considered as statistically significant.

## Results

Demographic and anthropometric data on our subjects subjected to normoxic and hypoxic training conditions are given in Table 1. These were middle‐aged overweight, untrained men. Under normoxic conditions, these values were 96 ± 1 and 95 ± 1% at the beginning and 96 ± 1 and 94 ± 1% at the end of training. Under hypoxic conditions, these values were 91 ± 1 and 85 ± 1%, and 93 ± 1 and 86 ± 1%, respectively. The saturations corresponded to the planned conditions.

**Table 1:** Anthropometric data before and after training under normoxia (*N* = 11) or hypoxia (*N* = 12)

	Normoxia		Hypoxia	
	Before	After	Before	After
Age (years)	57.6 ± 2.2		55.0 ± 2.1	**0.3**
Height (m)	1.79 ± 0.02		1.76 ± 0.02	
Weight (kg)	108.5 ± 3.0	106.2 ± 3.3^a^	109.1 ± 5.2	107.6 ± 5.3^a^
BMI (kg/m^2^)	34.1 ± 0.9	33.4 ± 1.1^a^	35.5 ± 1.4	34.9 ± 1.4^a^
Waist (W, cm)	117 ± 3	115 ± 3^a^	121 ± 3	119 ± 3^a^
Hip (H, cm))	115 ± 2	115 ± 2	116 ± 3	115 ± 3
W/H ratio	1.02 ± 0.01	1.00 ± 0.02	1.05 ± 0.02	1.03 ± 0.02
*BIA*				
FFM (%)	69.9 ± 2.3	70.0 ± 1.9	67.5 ± 1.8	68.3 ± 1.6
FM (%)	30.1 ± 2.3	30.0 ± 1.9	32.5 ± 1.8	31.7 ± 1.6
*BodPod*				
FFM (%)	64.7 ± 1.8	64.6 ± 1.9	63.7 ± 1.7	62.5 ± 2.0
FM (%)	35.3 ± 1.8	35.4 ± 1.9	36.3 ± 1.7	37.6 ± 2.0
REE (kcal/d)	1891 ± 99	1901 ± 45	1935 ± 95	1962 ± 98
RER	0.75 ± 0.01	0.78 ± 0.01	0.75 ± 0.01	0.76 ± 0.01

Data are given as Mean ± SE.

FFM, fat‐free mass; FM, fat mass; REE, resting energy expenditure; RER, respiratory exchange ratio ($\dot{V}$O_2_/$\dot{V}$CO_2_).

a *P* < 0.05 between visits.

Training reduced body mass index and waist circumference under both conditions. Heart rate, blood pressure, lipid and glucose values are reported in Table 2. Training lowered systolic blood pressure under both training conditions. LDL cholesterol, but not triglyceride, concentrations were significantly reduced by training. Table 3 demonstrates effects on workload, *V*O_2_max, resting energy expenditure, and lactate‐related parameters. Workload and heart rate under training showed modest effects after endurance training, albeit only under normoxia‐training conditions. We also measured cardiac dimensions with MRI and examined the expression of candidate marker genes in adipocytes under the various conditions with RT‐PCR, as shown in Tables 4 and 5. No consistent effects of training under either normoxic or hypoxic conditions were observed.

**Table 2:** Cardiometabolic risk factors before and after exercise training under normoxia (*N* = 11) or hypoxia (*N* = 12)

	Normoxia		Hypoxia	
	Before	After	Before	After
HR (bpm)	66 ± 2	66 ± 2	67 ± 3	61 ± 3
SBP (mmHg)	135 ± 4	123 ± 5^a^	145 ± 3	137 ± 3^a^
DPB (mmHg)	83 ± 3	77 ± 3	81 ± 2	79 ± 3
Glucose (mmol/L)	5.9 ± 0.4	5.9 ± 0.4	6.1 ± 0.5	5.9 ± 0.3
Insulin (*μ*U/mL)	19 ± 3	17 ± 4	15 ± 2	18 ± 3
HbA1c (%)	5.9 ± 0.2	5.8 ± 0.2	5.9 ± 0.3	5.7 ± 0.2
TG (mg/dL)	228 ± 26	188 ± 23	218 ± 59	163 ± 25
HDL (mg/dL)	43 ± 2	41 ± 2	52 ± 5	47 ± 4
LDL (mg/dL)	129 ± 14	114 ± 10^b^	147 ± 14	134 ± 14^b^
LDL/HDL	3.1 ± 0.3	3.0 ± 0.3	3.0 ± 0.3	3.0 ± 0.3

Data are given as Mean ± SE.

HR, heart rate; SBP/DBP, systolic/diastolic blood pressure; TG, triglycerides; HDL/LDL, high/low density lipoprotein.

a *P* < 0.05 between visits

b *P* < 0.01 between visits.

**Table 3:** Spiroergometry parameter before and after exercise training under normoxia (*N* = 9) or hypoxia (*N* = 12)

	Normoxia		Hypoxia	
	Before	After	Before	After
Workload_max_ (W)	158 ± 31	164 ± 28	152 ± 23	156 ± 30
HR_max_ (bpm)	132 ± 20	134 ± 23	141 ± 19	138 ± 15
*V*O_2_max (mL/min)	2434 ± 593	2507 ± 535	2318 ± 330	2376 ± 421
RER_max_	0.98 ± 0.04	0.98 ± 0.04	0.99 ± 0.05	0.99 ± 0.06
Lactate_max_ (mmol/L)	5.54 ± 2.16	5.43 ± 1.85	5.89 ± 1.65	5.40 ± 0.86
Lactate threshold (mmol/L)	1.60 ± 0.65	1.77 ± 0.66	1.69 ± 0.37	1.72 ± 0.32
Workload_Training_ (W)	81 ± 19	102 ± 28^a^	86 ± 19	93 ± 17
HR_Training_ (bpm)	102 ± 11	109 ± 13^a^	112 ± 14	110 ± 11

Data are given as Mean ± SD

HR, heart rate; RER, respiratory exchange ratio ($\dot{V}$CO_2_/$\dot{V}$O_2_).

a *P* < 0.05 between visits.

**Table 4:** Cardio‐MRT data before and after exercise training under normoxia (*N* = 6) or hypoxia (*N* = 8)

	Normoxia		Hypoxia	
	Before	After	Before	After
EDV (mL)	158.3 ± 11.5	161.1 ± 13.2	168.0 ± 31.1	171.3 ± 34.1
ESV (mL)	63.2 ± 9.0	62.5 ± 12.2	61.2 ± 17.9	64.0 ± 19.8
SV (mL)	95.0 ± 11.7	98.6 ± 5.8	106.8 ± 16.5	107.3 ± 15.5^a^
EF (%)	60.0 ± 5.6	61.5 ± 5.3	64.2 ± 5.6	63.5 ± 5.2
Myomass (g)	135.1 ± 19.8	138.6 ± 17.3	151.2 ± 33.2	150.7 ± 32.3
EDV/H (mL/m)	87.6 ± 5.8	83.3 ± 16.1	96.3 ± 17.1	97.9 ± 18.7
EDV/BSA (mL/m^2^)	69.6 ± 4.8	68.1 ± 7.9	76.4 ± 12.0	76.7 ± 13.8
ESV/H (mL/m)	34.5 ± 6.0	32.3 ± 9.0	34.1 ± 10.1	36.5 ± 11.0
ESV/BSA (mL/m^2^)	27.8 ± 4.1	26.4 ± 5.7	27.7 ± 7.2	28.6 ± 8.4
SV/H (mL/m)	52.6 ± 6.1	51.0 ± 9.1	61.2 ± 8.8^a^	61.4 ± 8.4^a^
SV/BSA (mL/m^2^)	41.3 ± 4.6	41.7 ± 4.4	48.9 ± 6.9^a^	48.2 ± 6.2^a^
Myomass/H (g/m)	74.7 ± 10.1	71.4 ± 16.7	86.6 ± 18.0	86.1 ± 17.4
Myomass/BSA (g/m^2^)	59.3 ± 7.8	58.7 ± 9.6	69.0 ± 15.4	67.4 ± 12.5
LVRI	0.86 ± 0.15	0.87 ± 0.13	0.91 ± 0.17	0.89 ± 0.14

Data are given as Mean ± SD

EDV, end‐diastolic volume; ESV, end‐systolic volume; SV, stroke volume; EF, ejection fraction; H, height; BSA, body surface area; LVRI, left ventricular remodeling index.

a *P* < 0.05, hypoxia versus normoxia at corresponding time points.

**Table 5:** Relative changes in adipocyte and myocellular mRNA expression of some marker genes after exercise training under either normoxic or hypoxic conditions

	Normoxia *N* = 8	Hypoxia *N* = 11
Adipose tissue		
HSL	4.5 ± 6.8	1.0 ± 1.1
PGC1a	1.2 ± 0.7	0.8 ± 0.5
Skeletal muscle		
Akt2	1.2 ± 0.5	1.2 ± 0.9
CPT1	1.3 ± 0.7	1.5 ± 0.9
Glut4	1.3 ± 0.9	1.4 ± 1.2
HIF1a	1.3 ± 0.6	1.0 ± 0.4
MEF2a	1.1 ± 0.4	1.0 ± 0.5
PGC1a	1.2 ± 0.5	1.1 ± 1.2
PGC1b	1.1 ± 0.2	1.0 ± 0.7
PRKAA1	1.1 ± 0.3	0.9 ± 0.4

Data are given as Mean ± SD.

HSL, hormone‐sensitive lipase; PGC 1 a/b, peroxisome proliferator activated receptor gamma coactivator 1 a/b; Akt2, RAC‐beta serine/threonine‐protein kinase/protein kinase B beta; CPT1, carnitine palmitoyl transferase 1; HIF1a, hypoxia‐inducible factor 1 alpha; MEF2a, myocyte enhancer factor 2a; PRKAA1, protein kinase AMP‐activated catalytic subunit alpha 1.

**Figure 2:**
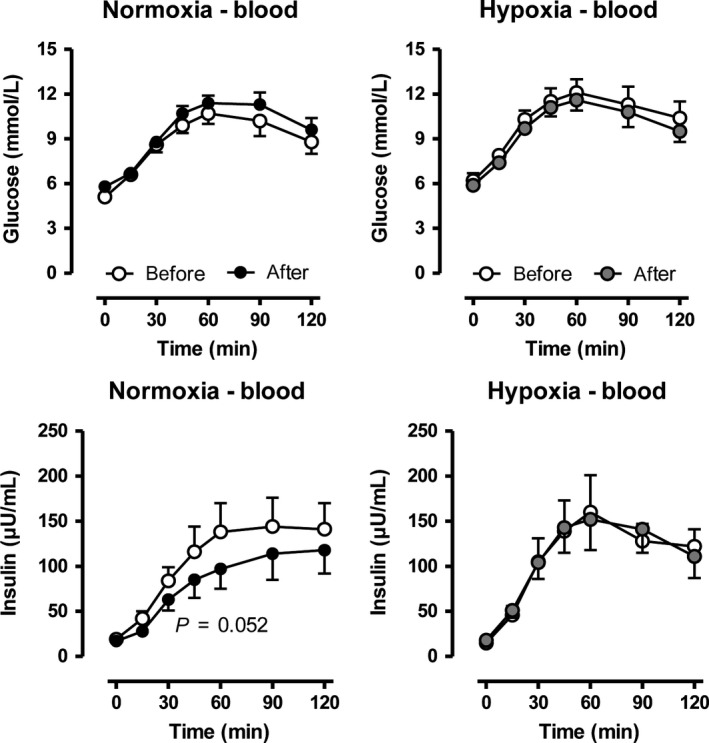
Changes in blood glucose and insulin levels after an oral glucose load before and after a moderate 6‐week training under either normobaric normoxic (*N* = 11) or hypoxic (*N* = 12) conditions. Data are given as means ± SE.

**Figure 3:**
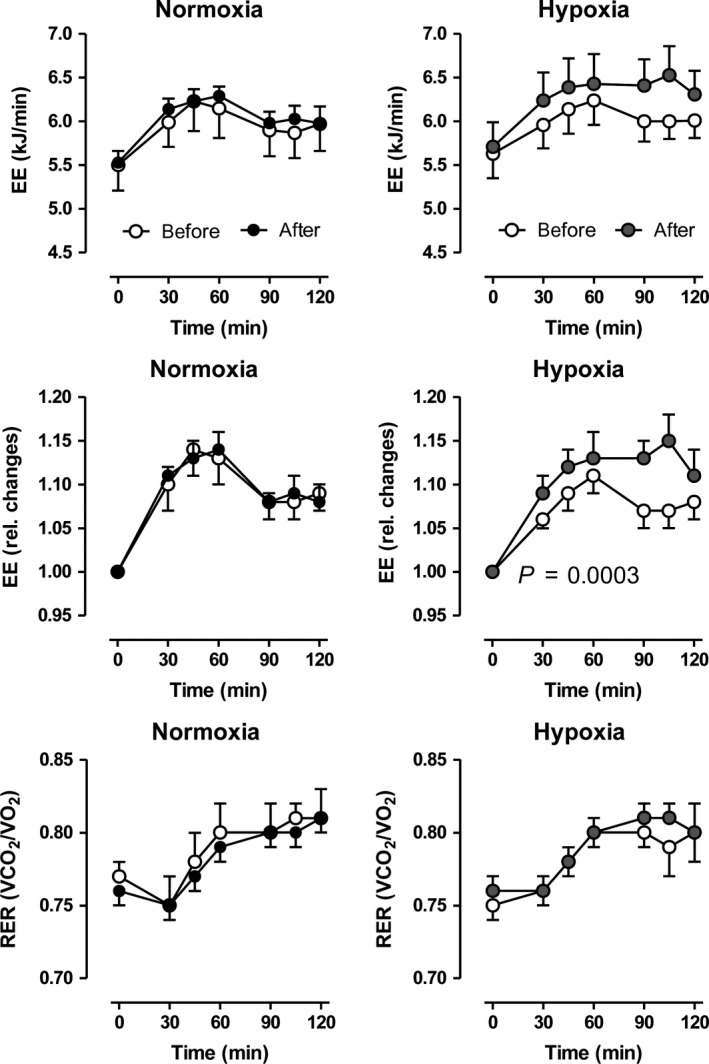
Changes in energy expenditure (absolute and relative) and respiratory exchange ratio (RER) after an oral glucose load (75 g) before and after a moderate 6‐week training under either normobaric normoxic (*N* = 11) or hypoxic (*N* = 12) conditions. Data are given as means ± SE.

The training effects on glucose tolerance were not different comparing normoxia to hypoxia. Insulin levels after the oral glucose load were marginally reduced after normoxic, but not after hypoxia‐training conditioning (Fig. 2). Postprandial energy expenditure, on the other hand was significantly higher after training under hypoxia (Fig. 3), although no effect on the respiratory exchange ratio was observed.

**Figure 4:**
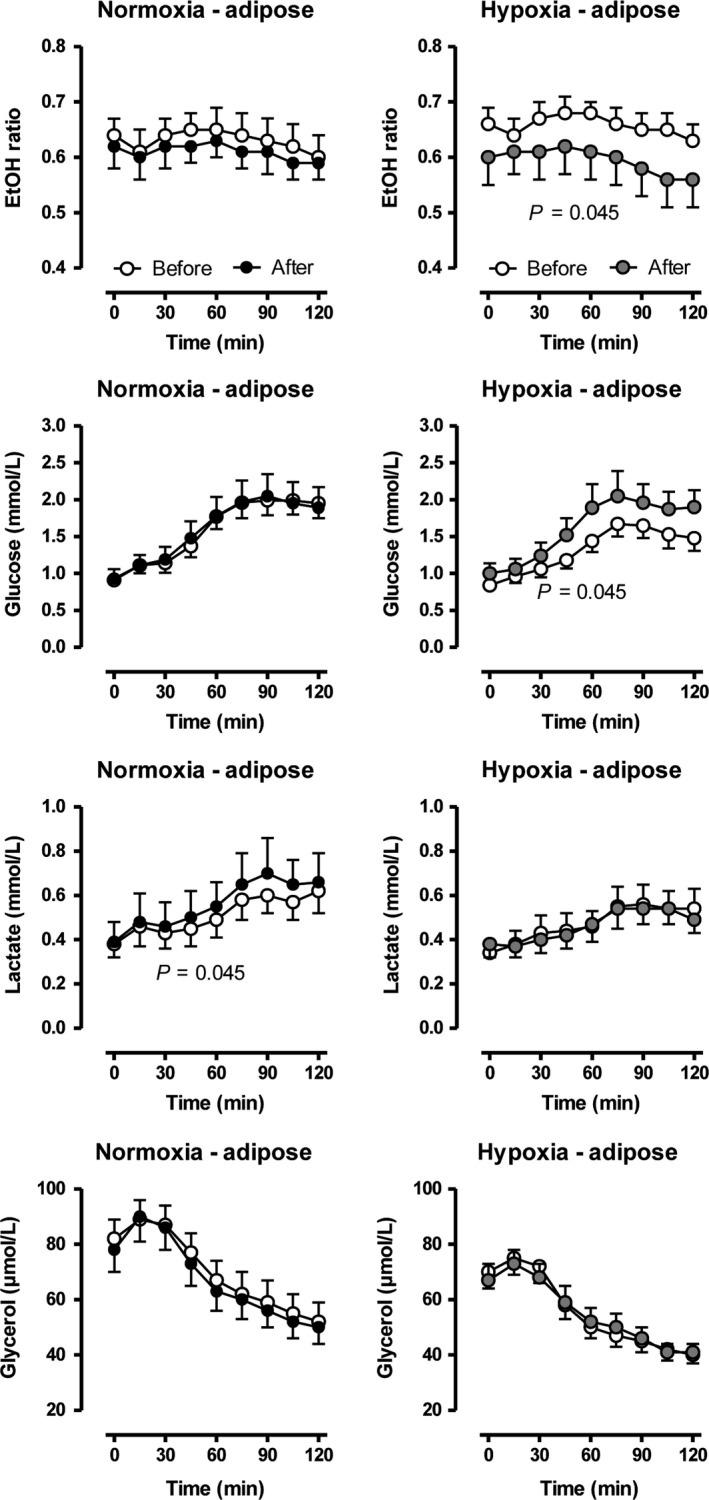
Changes in ethanol (EtOH) ratio and dialysate glucose, lactate and glycerol concentrations in adipose tissue after an oral glucose load before and after a moderate 6‐week training under either normobaric normoxic (*N* = 11) or hypoxic (*N* = 12) conditions. Data are given as means ± SE.

**Figure 5:**
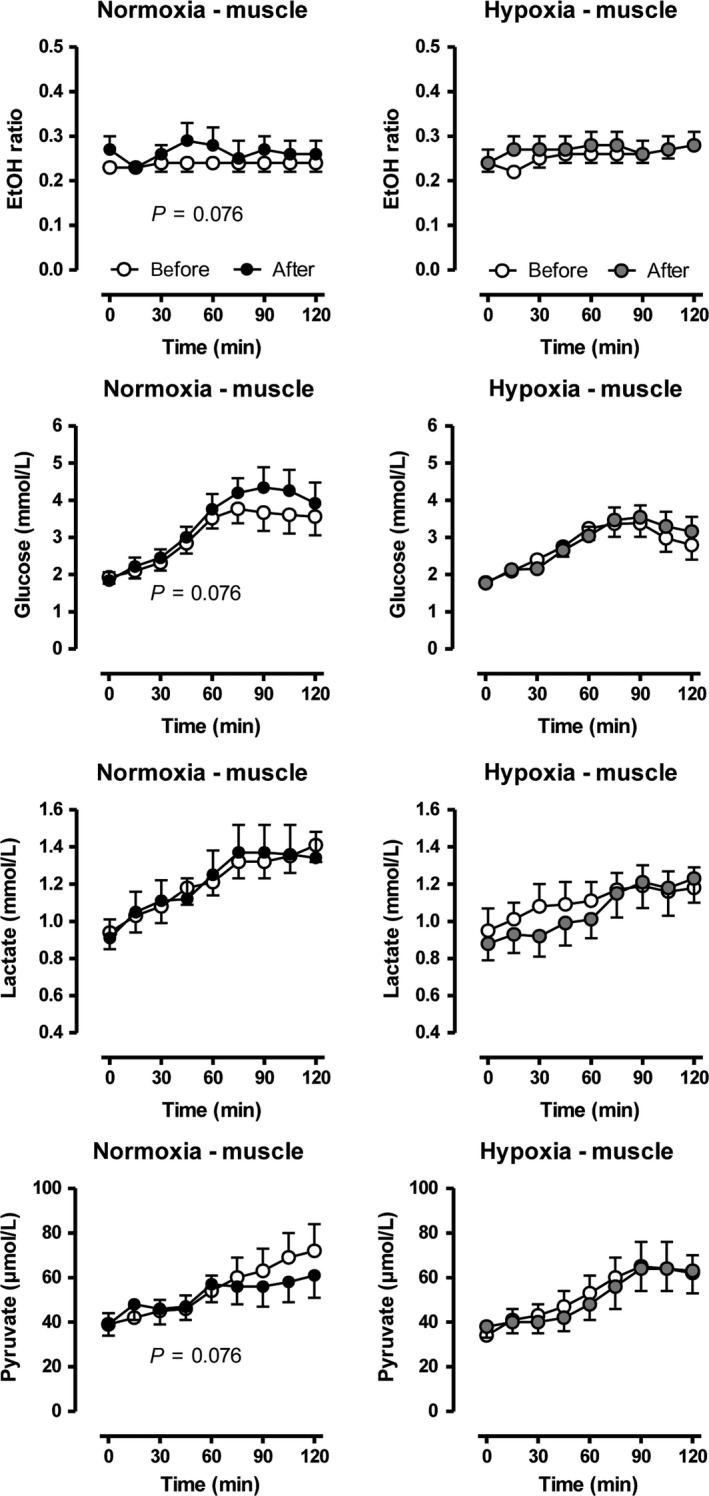
Changes in ethanol (EtOH) ratio and dialysate glucose, lactate and glycerol concentrations in skeletal muscle after an oral glucose load before and after a moderate 6‐week training under either normobaric normoxic (*N* = 11) or hypoxic (*N* = 12) conditions. Data are given as means ± SE.

The adipose‐tissue microdialysis data indicated that tissue perfusion, as reflected by the ethanol ratio, was increased after training under hypoxic conditions (Fig. 4). Similarly, dialysate glucose concentrations were mildly increased after training after hypoxia training. No effects were observed on either dialysate lactate or glycerol. The dialysate skeletal muscle data showed no effect of training or oxygen availability on ethanol ratio and dialysate glucose, lactate, and pyruvate (Fig. 5).

**Figure 6:**
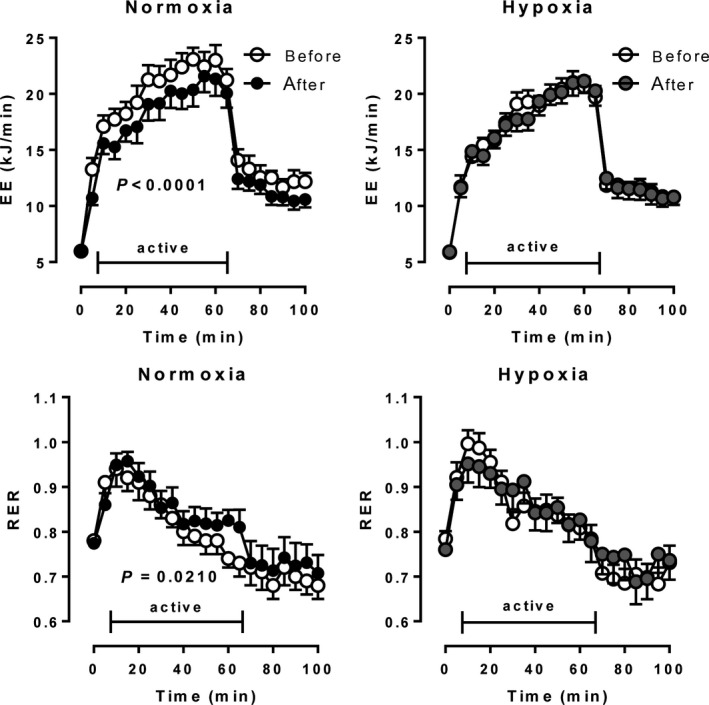
Changes in energy expenditure (EE) and respiratory exchange ratio (RER, $\dot{V}$ CO_2_/$\dot{V}$O_2_) after a 12 h overnight fast and moderate bicycle exercise over 60 min before and after a moderate 6‐week training under either normobaric normoxic (*N* = 11) or hypoxic (*N* = 12) conditions. Data are given as means ± SE.

During the 60 min exercise test in our metabolic chamber after a 12‐h overnight fast, EE increased about 3.8‐fold and 3.5‐fold (*P* < 0.05) in the normoxia and hypoxia groups, respectively, before the training program (Fig. 6). After the training program, this increase in EE was significantly lower in the normoxia group (3.5‐fold, *P* < 0.0001), but remained unchanged in the hypoxia group. Before the training program, the RER values increased in both groups after starting the exercise indicating increases in carbohydrate oxidation, followed by steady decreases indicating an increased fueling by fatty acid oxidation (Fig. 6). After the training, RERs changed in nearly the same way. However, at all time‐points, RER values were significantly higher in the normoxia group, but not in the hypoxia group, indicating a higher fueling by carbohydrate oxidation.

**Figure 7:**
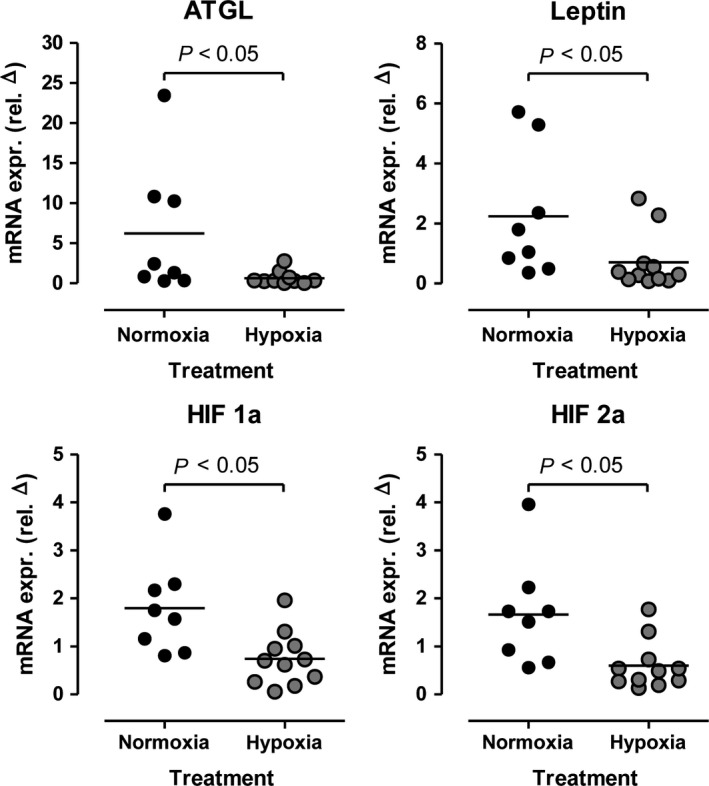
Relative changes in adipose tissue mRNA expression of adipose triglyceride lipase (ATGL), leptin, and hypoxia‐induced factor (HIF) 1alpha and 2alpha before and after a moderate 6‐week training under either normobaric normoxic (*N* = 8) or hypoxic (*N* = 11) conditions.

We measured gene expression in adipose tissue by determining mRNA of adipose triglyceride lipase (ATGL), leptin, and hypoxia‐induced factor (HIF) 1alpha and 2alpha. Here, significant effects were observed (Fig. 7). mRNA expression of all four genes was slightly but significantly increased after training under normoxic but not hypoxic conditions.

## Discussion

We found that men with metabolic syndrome benefit from a regulated exercise program. They lost weight, decreased their waist circumference and lowered their BMI values. Systolic blood pressure decreased as well, similar to a pharmacological intervention. Positive effects were observed on LDL cholesterol, but not triglyceride values. Our normoxia patients had a PaO_2_ slightly less than 100 mm Hg, while our hypoxia patients at the end of their sessions had a PaO_2_ slightly less than 60 mm Hg. This difference would place the hypoxia exercisers between 8000 and 10,000 feet, or between 2500 and 3000 m, compared to sea level. We could not show that exercising at these “altitudes” provides an additional added value for such patients. Undocumented claims, such as commercialization, in this direction should be abandoned and if not, then regulated by authorities.

Nevertheless, our study showed a beneficial effect of physical exercise on all parameters examined. The subjects, lost weight, the body‐mass parameters were improved, their blood pressures were reduced, and their serum lipid values were improved. With this protocol, blood sugar values were hardly influenced and hemoglobin A1C values showed solely a reduction trend. With the exercise sessions, serum lactate and pyruvate concentrations increased and almost doubled their resting values, although hypoxia training had no influence on these effects. Our training conditions and the degree of hypoxia that we induced were not near the notion of “dysoxia”, as a cause for lactate production. Investigators have substantially revised their ideas about lack of oxygen as responsible for lactate production during exercise (Ferguson et al. 2018). Originally regarded as a “hypoxic waste product”, many clinicians still view lactate in this manner. Rasmussen et al. (2001) have indicated that even when exercised repetitively to exhaustion, mitochondrial function remained unperturbed.

At the tissue level, we found a lower ethanol ratio in adipose tissue after training under hypoxia, both at baseline and after the glucose load, indicating an increased tissue perfusion. An increased tissue perfusion is not only associated with an improved substrate supply but also an increased product removal. Therefore, the real lactate and glycerol production rates are much higher than indicated by the dialysate concentrations. Higher tissue levels of lactate and glycerol indicate a more lipolytic state of adipose tissue (Szabo et al. 2013). Thus, training under hypoxia obviously improves adipose tissue perfusion and dynamics in metabolism.

In an earlier study, we tested 20 healthy men in a similar protocol (Haufe et al. 2008). Exercise capacity improved similarly with both interventions. With hypoxia training, body fat content, triglycerides, HOMA‐Index, fasting insulin, and area under the curve for insulin during the oral glucose tolerance test improved more than with the training in normoxia. We did not observe major changes in adipokine measurements. We were not able to show any benefits of hypoxia training here, although metabolic syndrome patients are not normal and are not able to deliver the same kinds of physical exertion. The idea that workload and, therefore, mechanic strain could be reduced under hypoxic conditions, which could be particularly beneficial in obese patients, is not corroborated here. We emphasize that our project was aimed at diseased patients; the idea that normobaric‐hypoxia training could be therapeutic was perhaps premature.

Previous studies from our group suggested that hypoxia and exercise may have a synergistic effect on cardiovascular and metabolic risk factors. Earlier, we conducted a single blind study in overweight to obese subjects to test the hypothesis that training under hypoxia as used here results in similar or even greater improvement in body weight and metabolic risk markers compared with exercise under normoxia (Wiesner et al. 2010). After an initial metabolic evaluation including incremental exercise testing, the subjects in that study trained in normoxic or hypoxic conditions thrice weekly over a 4‐week period at a heart rate corresponding to 65% of maximum oxygen uptake. In that study, we concluded that in obese subjects, training in hypoxia elicits a similar or even better response in terms of physical fitness, metabolic risk markers, and body composition at a lower workload. These results gave us the impetus to test the current hypotheses. The current results do not inspire confidence in the earlier conclusion. Possibly, the additional confounder of the “metabolic syndrome” in the current investigation could not be overcome by the intervention.

Normobaric hypoxic conditioning to maximize weight loss and ameliorate cardio‐metabolic health in both animals and man has been extensively reviewed (Hobbins et al. 2017). Exercise sessions lasted from 5 days up to 8 months in the studies cited. Reductions in insulin, body weight, and blood pressure were reported. We observed all these effects in the study reported here. Unfortunately, hypoxic conditioning did not influence any of these parameters that took place with sea‐level training as well. Hobbins et al. (2017) indicated that proving a beneficial effect on overall fitness through hypoxic condition, compared to sea‐level training is difficult. In some earlier studies, the workload under the two conditions was not the same. Morishima et al. (2014) studied 20 sedentary subjects under similar conditions tested here. They performed a 4‐week training at 55% of maximal oxygen uptake expressed as *V*O_2_max for each condition. Before and after the training period, *V*O_2_max, whole body fat mass, abdominal fat area, intramyocellular lipid content, fasting and postprandial appetite‐related hormonal responses were determined. Both groups showed a significant increase in *V*O_2_max following training. Hypoxic training for 4 weeks resulted in greater improvement in glucose tolerance without loss of whole body fat mass, abdominal fat area or intramyocellular lipid content. We observed similar improvements in our subjects under both conditions.

An unexpected finding in our study was the increase in adipose triglyceride lipase, leptin, and particularly HIF 1‐alpha as well as HIF 2‐alpha mRNAs in response to normoxic, but not hypoxic training conditions. We would have doubted the adipose‐tissue HIF results; however, the skeletal muscle biopsies we performed gave HIF results that were in the same direction, a 30% increase under normoxic versus hypoxic training conditions, albeit not statistically significant. HIF has been identified as a master regulator for the expression of genes involved in the hypoxia response, such as genes coding for glucose transporters, glycolytic enzymes and vascular endothelial growth factor (VEGF) (Hoppeler et al. 2003). Within the hypoxic cells, the diminished level of oxygen stabilizes the HIF‐1*α* protein and activates the HIF‐dependent transcription leading to the regulation of the expression of numerous genes. Although hypoxia is the main stimulator of HIFs activity, also in normoxic conditions numerous stimuli, such as reactive oxygen species (ROS), metal ions, and growth factors may influence HIF levels. The formation of reactive oxygen species (ROS) occurs in mitochondria during oxidative phosphorylation. How exactly ROS could interfere with HIF‐1alpha as well as MAP kinase and other signaling pathways is unknown. We did not measure ROS in our studies and whether or not ROS could have been increased by hypoxia training is unknown. Contractile activity in muscle alone should activate transcription factors including HIF (Lima et al. 2009). We did not perform longitudinal biopsies.

Our study has limitations. Our goal was to investigate both men and women. However, we failed in our efforts to recruit sufficient numbers of female subjects. We cannot speculate whether or not our intervention would have functioned differently in women. Our level of exercise intensity was perhaps not sufficient to bring about a more dramatic effect or to draw out the advantages of hypoxic training. An underestimated factor in achieving weight loss could be related to psychological behaviors. Exercising requires motivation and enjoyment. Our earlier study seemed to give more encouraging results for hypoxia training, albeit the differences were small (Wiesner et al. 2010). Furthermore, according to the calorimetric data during exercise, volunteers seemed to be initially more fit in the hypoxia compared to the normoxia group. Finally, we did not include an untreated control group. Human studies necessarily have some drawbacks since the subjects can subjected to being assigned.

Active hypoxic physical conditioning added little to active physical conditioning under sea‐level conditions in our study. The industrial and technological advancement, including miniaturized equipment, reduced costs, increased accessibility to environmental chambers, not to mention the continually advancing marketing techniques by entrepreneurs will undoubtedly move the hypoxia training business forward. Exercise training is a method‐of‐thought, a daily activity of habit. We suggest it is the training that counts, and far less so the FiO_2_. Nonetheless, we intend to continue our studies.

## Conflict of Interest

The authors have nothing to disclose.
